# Association between life’s essential 8 and biological ageing among US adults

**DOI:** 10.1186/s12967-023-04495-8

**Published:** 2023-09-14

**Authors:** Ronghuai Zhang, Min Wu, Wei Zhang, Xuna Liu, Jie Pu, Tao Wei, Zhanfang Zhu, Zhiguo Tang, Na Wei, Bo Liu, Qianwei Cui, Junkui Wang, Fuqiang Liu, Ying Lv

**Affiliations:** 1 Department of Cardiology, Shaanxi Provincial People’s Hospital, No. 256, Youyixi Road, Xi’an, 710068 Shaanxi China; 2 Shaanxi Provincial Key Laboratory of Infection and Immune Diseases, Shaanxi Provincial People’s Hospital, Xi’an, People’s Republic of China; 3 Department of Gastroenterology, The Second Affiliated Hospital of Xi’an Jiaotong University, Xi’an 710004, China; 4 Department of Cardiovascular Surgery, Shaanxi Provincial People’s Hospital, Xi’an, People’s Republic of China; 5 Xi’an Jiaotong University Hospital, Xi’an, People’s Republic of China

**Keywords:** Life’s Essential 8, Biological Ageing, NHANES, Cardiovascular Health

## Abstract

**Background:**

Biological ageing is tightly linked to cardiovascular disease (CVD). We aimed to investigate the relationship between Life’s Essential 8 (LE8), a currently updated measure of cardiovascular health (CVH), and biological ageing.

**Methods:**

This cross-sectional study selected adults ≥ 20 years of age from the 2005–2010 National Health and Nutrition Examination Survey. LE8 scores (range 0–100) were obtained from measurements based on American Heart Association definitions, divided into health behavior and health factor scores. Biological ageing was assessed by different methods including phenotypic age, phenotypic age acceleration (PhenoAgeAccel), biological age and biological age acceleration (BioAgeAccel). Correlations were analyzed by weighted linear regression and restricted cubic spline models.

**Results:**

Of the 11,729 participants included, the mean age was 47.41 ± 0.36 years and 5983 (51.01%) were female. The mean phenotypic and biological ages were 42.96 ± 0.41 and 46.75 ± 0.39 years, respectively, and the mean LE8 score was 67.71 ± 0.35. After adjusting for potential confounders, higher LE8 scores were associated with lower phenotypic age, biological age, PhenoAgeAccel, and BioAgeAccel, with nonlinear dose–response relationships. Negative associations were also found between health behavior and health factor scores and biological ageing, and were stronger for health factors. In health factor-specific analyses, the β negativity was greater for blood glucose and blood pressure. The inverse correlations of LE8 scores with phenotypic age and biological age in the stratified analyses remained solid across strata.

**Conclusions:**

LE8 and its subscale scores were strongly negatively related to biological ageing. Encouraging optimal CVH levels may be advantageous in preventing and slowing down ageing.

**Supplementary Information:**

The online version contains supplementary material available at 10.1186/s12967-023-04495-8.

## Introduction

Population ageing has turned into a global phenomenon, with 9% of the world's population currently over the age of 65, a figure that is predicted to rise to 16% by 2050, according to a United Nations report in 2019 [1]. Ageing imposes an unbearable burden of chronic disease on humanity, resulting in significant social and economic costs [2]. Ageing is influenced by a range of environmental, biopsychosocial and sociodemographic factors, all of which may act synergistically to determine the ageing process [3]. In addition, ageing contributes to the onset and development of age-related diseases, particularly cardiovascular diseases, [4] while age-related diseases can also accelerate ageing. A large number of previous studies have revealed that ageing promotes the development and onset of a wide range of age-related diseases, particularly cardiovascular disease [5–8]. However, little research has been conducted on whether maintaining cardiovascular health can delay ageing.

In 2010, the American Heart Association (AHA) released the Life's Simple 7 (LS7) a measure of cardiovascular health (CVH), which includes a well-balanced diet, non-smoking, a healthy body mass index (BMI), reasonable level of physical activity, blood pressure, fasting blood glucose, and total cholesterol, to better promote health for the population [9]. A previous study has found a negative correlation between LS7 and epigenetic age acceleration, suggesting that ideal CVH is associated with a longer lifespan. Recently, to better assess CVH, the AHA introduced Life’s Essential 8 (LE8), which featured important updates to incorporate sleep quality indicators and upgrade scoring algorithms compared to the original LS7 [10]. The LE8 is a scoring system that is more sensitive to inter-individual differences and emphasis the social determinants of health and mental health that maintain or improve CVH [11]. There are currently no studies correlating LE8 with biological ageing.

Ageing is a complex biological process that involves multiple dimensions of cells, tissues, and organs, [3] so there are various ways to reflect biological ageing, such as phenotypic age, biological age, leukocyte telomere length, and metabolic age score [6, 12–14]. In this study, phenotypic age, biological age, phenotypic age acceleration (PhenoAgeAccel), and biological age acceleration (BioAgeAccel) were chosen to reflect ageing. In general, phenotypic age corresponds to chronological age at the same risk of death, biological age refers to chronological age at the same physiological function, and accelerated phenotypic age and accelerated biological age further quantify the difference between them and chronological age. Phenotypic age and biological age calculated based on clinically observable data are considered to be more reliable predictors of ageing outcomes. Therefore, this study aimed to estimate the correlation between LE8 and biological ageing using the National Health and Nutrition Examination Surveys (NHANES) data.

## Method

### Study population

This cross-sectional study included participants from the nationally representative consecutive NHANES 2005–2010. These years were chosen because sleep data in LE8 began to be collected in 2005 and C-reactive protein (CRP), which was used to calculate phenotypic age and biological age, was collected from 1999 to 2010. NHANES was approved by the National Center for Health Statistics' ethics review committee, and all participants submitted written informed consent. All procedures for this study were conducted in accordance with relevant guidelines and regulations (https://www.cdc.gov/nchs/data_access/restrictions.htm). Of the 31,034 subjects in the NHANES 2005–2010, individuals were excluded if (1) they were under 20 years of age (n = 13,920), (2) they had missing data on LE8 (n = 4229), (3) they had missing values regarding phenotypic age and biological age (n = 1156), (4) they had any missing values for marital and educational status (n = 18). Ultimately, a total of 11,729 subjects were included in this research (Additional file 1: Fig. S1).

### Measurement of LE8

The LE8 score comprises 4 health behaviors (diet, physical activity, nicotine exposure, and sleep duration) and 4 health factors (BMI, non-high-density lipoprotein cholesterol/non–HDL cholesterol, blood glucose, and blood pressure). Dietary indicators were assessed using the Healthy Eating Index (HEI) 2015 measured by the subjects' 24-h dietary review [15]. Physical activity, nicotine exposure, sleep data, diabetes history, and medication history were collected through a self-report questionnaire. Height, weight, and blood pressure were measured by physical examination. BMI was obtained by dividing weight (kilograms) by the square of height (meters). Non–HDL cholesterol, plasma glucose, and hemoglobin A1c were measured from collected blood samples. The detailed algorithm to calculate the LE8 scores for the indicators in the NHANES data has been published previously (Additional file 1:Table S1), in which each of the 8 CVH indicators was scored on a scale ranging from 0 to 100, and the total LE8 score was calculated as an unweighted average of the 8 indicators [10, 11]. Meanwhile, participants with high CVH were considered to have LE8 scores of 80–100; moderate CVH was 50–79; and low CVH was 0–49 [10]. In addition, our study adopted the same cut-off points to classify health behavior and health factor scores to further investigate the relationship between the LE8 subscales and biological ageing.

### Measurement of biological ageing markers

Biological ageing was measured using phenotypic age and biological age, which use different biomarkers and different ways of calculation. Phenotypic age was calculated using the following formula, in which, xb = − 19.907 − 0.0336 × Albumin + 0.0095 × Creatinine + 0.1953 × Glucose + 0.0954 × LnCRP − 0.0120 × Lymphocyte Percentage + 0.0268 × Mean Cell Volume + 0.3306 × Erythrocyte Distribution Width + 0.00188 × Alkaline Phosphatase + 0.0554 × Leukocyte Count + 0.0804 × chronological age [13].

$$ {\text{Phenotypic}}\;{\text{age}}\; = \;141.50 + \frac{{{\text{Ln[}} - {0}{\text{.00553}} \times {\text{Ln}}\left( {{\text{exp}}\left( {\frac{{{(} - {1}{\text{.51714}} \times {\text{exp(xb)}}}}{0.0076927}} \right)} \right){]}}}{0.09165} $$

Klemera presented biological age based on eight biomarkers (Ln-CRP, serum creatinine, glycated hemoglobin, serum albumin, serum total cholesterol, serum urea nitrogen, serum alkaline phosphatase, and systolic blood pressure) [12, 16]. The number of biomarkers and samples were indicated by j and i values, respectively. The slope, intercept, and root mean square error of the regression of biomarkers with chronological age are presented by k, q, and s values, respectively. The variance explained by the regression of biomarkers against chronological age is plotted as r_j_^2^.

$$ {\text{BA}}_E \; = \;\frac{{\sum_{j\; = \;1}^m {\left( {x_j , - ,q_j } \right)\left( {\frac{k_j }{{s_j^2 }}} \right)} }}{{\sum_{j\; = \;1}^m {\left( {\frac{k_j }{{s_j^2 }}} \right)^2 } }} $$

$$ {\text{r}}_{char} \; = \;\frac{{\sum_{j\; = \;1}^m {\frac{r_j^2 }{{\sqrt {1 - r_j^2 } }}} }}{{\sum_{j\; = \;1}^m {\frac{r_j }{{\sqrt {1 - r_j^2 } }}} }} $$

$$ \begin{aligned} s_{{\text{BA}}}^2 \; = & \;\frac{{\sum_{j\; = \;1}^n {\left( {\left( {{\text{BA}}_{Ei} , - ,{\text{CA}}_i } \right), - ,\frac{{\sum_{i\; = \;1}^n {\left( {{\text{BA}}_{Ei} , - ,{\text{CA}}_i } \right)} }}{n}} \right)} }}{n} \\ - & \;\left( {\frac{{{1} - {\text{r}}_{char}^2 }}{{{\text{r}}_{char}^2 }}} \right) \times \left( {\frac{{\left( {{\text{CA}}_{\max } , - ,{\text{CA}}_{\min } } \right)^2 }}{12m}} \right) \\ \end{aligned} $$

$$ {\text{Biological}}\;{\text{age}}\;{ = }\;\frac{{\sum_{j\; = \;1}^m {\left( {x_j , - ,q_j } \right)\left( {\frac{k_j }{{s_j^2 }}} \right)\; + \frac{{{\text{CA}}}}{{s_{{\text{BA}}}^2 }}} }}{{\sum_{j\; = \;1}^m {\left( {\frac{k_j }{{s_j^2 }}} \right)^2 \; + \;\frac{1}{{s_{{\text{BA}}}^2 }}} }} $$

In addition, we calculated the difference between phenotypic age, biological age, and chronological age separately and obtained PhenoAgeAccel and biological age BioAgeAccel [17]. PhenoAgeAccel and BioAgeAccel were used to identify whether an individual's phenotypic age and biological age were less than or greater than their chronological age, with their negative values indicating that the individual's phenotypic age and biological age were younger.

### Covariate assessment

In our study, covariates consisted of several factors previously displayed or assumed to be associated with LE8 or biological ageing, including age, sex (female, male), race (Mexican American, non-Hispanic black, non-Hispanic white, other Hispanic, other race), education (< high school, high school, > high school), marital status (divorced/separated/widowed, married/living with a partner, never married), and poverty-to-income ratio (PIR) (< 1.3, 1.3–3.5, > 3.5, no record), alcohol use (never, former, now, no record), hypertension, diabetes mellitus (DM), CVD, and cancer, where hypertension and DM were diagnosed through index measurements, medication use and self-reporting, and CVD and cancer were identified through self-reporting.

### Statistical analysis

Due to the complexity of the NHANES sampling design, appropriate weights were used for the sample analysis. For baseline characterization, weighted means (standard errors) were used for continuous variables, and sample sizes (weighted percentages) were used for categorical variables. To check for differences in variable characteristics between the different low- moderate-high CVH groups, ANOVA was used for differences in weighted means for continuous variables and the Rao—Scott χ2 test for differences in weighted percentages for categorical variables.

Weighted linear regression was used to analyze the association of LE8 score with biological ageing, (incorporating phenotypic age, biological age, PhenoAgeAccel, and BioAgeAccel), as well as the relationship between different degrees of CVH and biological ageing. Crude models did not adjust for any potential confounders. Model 1 adjusted for age, sex, race, education, marital status, PIR, and alcohol use. Model 2 was further adjusted for comorbidities, namely hypertension, CVD, diabetes, and cancer. In addition, the correlations between health behavior, health factor scores, and each of the LE8 scores with biological ageing were explored using weighted linear regression analyses, after adjusting for all confounding variables. Restricted cubic spline (RCS) was also used to further validate the link between LE8 scores and biological ageing. To examine different subpopulations at baseline, analyses were stratified by gender, age group, race, education, marriage, PIR, alcohol use, hypertension, CVD, diabetes, and cancer. The interaction of stratification factors with LE8 scores was investigated by multiplicative interaction tests, and the interaction between health behaviors score and health factors score was calculated in the same way. For sensitivity analyses, we excluded the individuals with comorbidity (including hypertension, CVD, diabetes, and cancer) to assess the robustness of our findings.

Statistical analyses for this study were carried out using R version 4.2.1 (R Foundation for Statistical Computing, Vienna, Austria; http://www.r-project.org) and statistical significance was ascertained by a two-sided P value of < 0.05.

## Results

### Baseline characteristics

Table 1 displayed the baseline characteristics of individuals grouped by low, moderate, and high CVH: of the 11,729 subjects, 51.01% were female, with a mean age of 47.41 ± 0.36 years, the majority were non-Hispanic whites (51.73%), and those with low, moderate, and high CVH numbered 1,546 (13.18%), 8,136 (69.37%), and 2,047 (17.45%), respectively. For demographic sociology, participants in the high CVH group were younger, more female, more white, more educated, less divorced/separated/widowed, wealthier, and less likely to consume alcohol than those in the low CVH group. In terms of comorbidities, high CVH participants were less likely to have hypertension, CVD, and diabetes, and there were no differences in cancer prevalence. Regarding biological ageing, better CVH had younger phenotypic age and biological age and had greater PhenoAgeAccel and BioAgeAccel.

**Table 1:** Baseline Characteristics of the study population

Variable	Total	Low	Moderate	High	P value
No. of participants	11,729	1546 (13.18)	8136 (69.37)	2047 (17.45)	
Age, y, mean (SE)	47.41 (0.36)	53.25 (0.61)	48.16 (0.35)	41.92 (0.54)	< 0.0001
Age, n (%)					< 0.0001
18–39	3744 (31.92)	233 (18.32)	2482 (33.49)	1029 (48.79)	
40–59	3965 (33.81)	596 (45.96)	2757 (40.80)	612 (36.48)	
≥ 60	4020 (34.27)	717 (35.71)	2897 (25.71)	406 (14.72)	
Sex, n (%)					< 0.0001
Female	5983 (51.01)	791 (52.82)	3934 (48.77)	1258 (62.04)	
Male	5746 (48.99)	755 (47.18)	4202 (51.23)	789 (37.96)	
Race, n (%)					< 0.0001
Mexican American	2084 (17.77)	216 (6.13)	1503 (8.07)	365 (7.41)	
Non-Hispanic Black	2155 (18.37)	431 (16.62)	1500 (10.08)	224 ( 5.53)	
Non-Hispanic White	6067 (51.73)	744 (69.51)	4175 (72.77)	1148 (75.96)	
Other Hispanic	977 (8.33)	115 (3.78)	664 (4.15)	198 (4.59)	
Other Race	446 (3.8)	40 (3.95)	294 (4.92)	112 (6.52)	
Education level, n (%)					< 0.0001
< High School	1328 (11.32)	255 (10.28)	935 ( 5.72)	138 ( 3.06)	
High School	4617 (39.36)	795 (51.84)	3331 (38.18)	491 (18.84)	
> High school	5784 (49.31)	496 (37.87)	3870 (56.10)	1418 (78.10)	
Marital status, n (%)					< 0.0001
Divorced/Separated/Widowed	2605 (22.21)	516 (30.64)	1820 (18.32)	269 (10.75)	
Married/Living with a partner	7275 (62.03)	857 (58.43)	5121 (67.41)	1297 (66.30)	
Never married	1849 (15.76)	173 (10.93)	1195 (14.27)	481 (22.95)	
Poverty-to-income ratio, n (%)					< 0.0001
< 1.3	3092 (26.36)	571 (27.04)	2113 (17.01)	408 (11.84)	
1.3–3.5	4226 (36.03)	566 (39.24)	3003 (35.46)	657 (29.02)	
> 3.5	3581 (30.53)	298 (27.50)	2440 (42.01)	843 (53.91)	
No record	830 (7.08)	111 (6.22)	580 (5.52)	139 (5.23)	
Alcohol status, n (%)					< 0.0001
Never	1461 (12.46)	187 ( 9.76)	969 ( 9.60)	305 (12.17)	
Former	2251 (19.19)	470 (28.14)	1577 (16.59)	204 ( 8.34)	
Now	7523 (64.14)	824 (58.02)	5254 (70.28)	1445 (75.27)	
No record	494 (4.21)	65 (4.07)	336 (3.54)	93 (4.22)	
Hypertension					< 0.0001
No	6882 (58.68)	419 (31.55)	4676 (61.46)	1787 (88.76)	
Yes	4847 (41.32)	1127 (68.45)	3460 (38.54)	260 (11.24)	
DM					< 0.0001
No	9940 (84.75)	921 (65.43)	7011 (89.71)	2008 (98.37)	
Yes	1789 (15.25)	625 (34.57)	1125 (10.29)	39 ( 1.63)	
CVD					< 0.0001
No	10,402 (88.69)	1166 (79.05)	7269 (91.71)	1967 (97.06)	
Yes	1327 (11.31)	380 (20.95)	867 ( 8.29)	80 ( 2.94)	
Cancer					0.13
No	10,583 (90.23)	1377 (89.23)	7328 (90.74)	1878 (91.55)	
Yes	1146 (9.77)	169 (10.77)	808 ( 9.26)	169 ( 8.45)	
Phenotypic age	42.96 (0.41)	54.34 (0.69)	43.83 (0.38)	34.22 (0.56)	< 0.0001
Biological age	46.75 (0.39)	57.60 (0.65)	47.64 (0.39)	38.26 (0.47)	< 0.0001
PhenoAgeAccel	− 4.46 (0.10)	1.08 (0.26)	− 4.33 (0.11)	− 7.71 (0.14)	< 0.0001
BioAgeAccel	− 0.66 (0.12)	4.35 (0.26)	− 0.53 (0.13)	− 3.67 (0.18)	< 0.0001
LE8 scores	67.71 (0.35)	42.35 (0.17)	65.97 (0.16)	86.49 (0.18)	< 0.0001
Health behaviors score	64.61 (0.47)	37.85 (0.59)	62.92 (0.33)	83.96 (0.33)	< 0.0001
HEI-2015 diet score	39.18 (0.66)	20.96 (0.82)	35.49 (0.56)	60.70 (0.84)	< 0.0001
Physical activity score	67.45 (0.71)	26.89 (1.38)	66.79 (0.79)	90.50 (0.69)	< 0.0001
Nicotine exposure score	69.34 (0.75)	39.38 (1.39)	67.24 (0.70)	91.70 (0.77)	< 0.0001
Sleep health score	82.49 (0.37)	64.17 (0.87)	82.18 (0.36)	92.95 (0.41)	< 0.0001
Health factors score	70.81 (0.31)	46.86 (0.45)	69.02 (0.24)	89.02 (0.28)	< 0.0001
Body mass index score	62.88 (0.57)	35.00 (1.18)	60.14 (0.44)	86.24 (0.63)	< 0.0001
Blood lipids score	62.35 (0.36)	40.69 (1.04)	59.75 (0.40)	82.02 (0.50)	< 0.0001
Blood glucose score	87.62 (0.32)	64.76 (0.90)	87.97 (0.30)	98.26 (0.26)	< 0.0001
Blood pressure score	70.38 (0.49)	46.98 (0.96)	68.22 (0.53)	89.57 (0.55)	< 0.0001

LE8, life’s essential 8; HEI, healthy eating index; DM, diabetes mellitus; CVD, cardiovascular disease; PhenoAgeAccel, phenotypic age acceleration; BioAgeAccel, biological age acceleration;

Data were presented as weighted percentages or means (95% confidence intervals);

Low CVH (cardiovascular health) was defined as a LE8 score of 0 to 49, moderate CVH of 50–79, and high CVH of 80–100

### Association between LE8/CVH and biological ageing

As presented in Table 2, weighted linear regression revealed negative associations between LE8 as well as CVH and biological ageing. In the analyses on LE8 and biological ageing, after adjusting for all confounding variables, LE8 was negatively associated with phenotypic age, biological age, PhenoAgeAccel, and BioAgeAccel (all P < 0.0001 in Model 2). Analyses on CVH and biological ageing showed statistically significant dose-decreasing trends for phenotypic age, biological age, accelerated phenotypic age, and accelerated biological age in the low-moderate-high CVH group (all P for trend < 0.0001), and the trend remained relatively stable across models. During the sensitivity analyses excluding comorbidities, the same trend was noted where phenotypic age and biological age were negatively correlated with LE8 and CVH (P for trend < 0.0001) (Additional file 1:Table S2).

**Table 2:** Weighted linear regression showing the relationship between LE8/CVH and biological ageing

	Crude model		Model 1		Model 2	
	β (95%CI)	P	β (95%CI)	P	β (95%CI)	P
	Phenotypic age					
LE8	− 0.450 (− 0.487, − 0.413)	< 0.0001	− 0.170 (− 0.181, − 0.158)	< 0.0001	− 0.129 (− 0.142, − 0.117)	< 0.0001
CVH						
Low	ref		ref		ref	
Moderate	− 10.505 (− 11.744, − 9.267)	< 0.0001	− 4.730 (− 5.325, − 4.135)	< 0.0001	− 3.047 (− 3.596, − 2.498)	< 0.0001
High	− 20.118 (− 21.734, − 18.502)	< 0.0001	− 7.406 (− 7.953, − 6.858)	< 0.0001	− 5.268 (− 5.831, − 4.705)	< 0.0001
P for trend		< 0.0001		< 0.0001		< 0.0001
	Biological age					
LE8	− 0.435 (− 0.465, − 0.405)	< 0.0001	− 0.187 (− 0.197, − 0.177)	< 0.0001	− 0.117 (− 0.127, − 0.107)	< 0.0001
CVH						
Low	ref		ref		ref	
Moderate	− 9.966 (− 11.172, − 8.760)	< 0.0001	− 5.101 (− 5.628, − 4.573)	< 0.0001	− 2.94 (− 3.381, − 2.498)	< 0.0001
High	− 19.347 (− 20.765, − 17.930)	< 0.0001	− 8.098 (− 8.697, − 7.498)	< 0.0001	− 4.76 (− 5.303, − 4.217)	< 0.0001
P for trend		< 0.0001		< 0.0001		< 0.0001
	PhenoAgeAccel					
LE8	− 0.193 (− 0.204, − 0.182)	< 0.0001	− 0.170 (− 0.181, − 0.158)	< 0.0001	− 0.129 (− 0.142, − 0.117)	< 0.0001
CVH						
Low	ref		ref		ref	
Moderate	− 5.413 (− 6.007, − 4.819)	< 0.0001	− 4.73 (− 5.325, − 4.135)	< 0.0001	− 3.047 (− 3.596, − 2.498)	< 0.0001
High	− 8.788 (− 9.312, − 8.264)	< 0.0001	− 7.406 (− 7.953, − 6.858)	< 0.0001	− 5.268 (− 5.831, − 4.705)	< 0.0001
P for trend		< 0.0001		< 0.0001		< 0.0001
	BioAgeAccel					
LE8	− 0.178 (− 0.189, − 0.166)	< 0.0001	− 0.187 (− 0.197, − 0.177)	< 0.0001	− 0.117 (− 0.127, − 0.107)	< 0.0001
CVH						
Low	ref		ref		ref	
Moderate	− 4.874 (− 5.429, − 4.320)	< 0.0001	− 5.101 (− 5.628, − 4.573)	< 0.0001	− 2.94 (− 3.381, − 2.498)	< 0.0001
High	− 8.017 (− 8.655, − 7.379)	< 0.0001	− 8.098 (− 8.697, − 7.498)	< 0.0001	− 4.76 (− 5.303, − 4.217)	< 0.0001
P for trend		< 0.0001		< 0.0001		< 0.0001

Crudel model: unadjusted model;

Model 1: Adjusted for age, sex, race, marital status, education, poverty-income ratio, and alcohol using;

Model 2: Additionally adjusted for hypertension, CVD, diabetes, and cancer

LE8, life’s essential 8; CVH, cardiovascular health; PhenoAgeAccel, phenotypic age acceleration; BioAgeAccel, biological age acceleration

Low CVH was defined as a LE8 score of 0 to 49, moderate CVH of 50–79, and high CVH of 80–100

### Relationship of health behaviors/health factors with biological ageing

Table 3 presented the results of linear regression of health behavior scores and health factor scores with phenotypic age and biological age. Health behavior scores and health factor scores were negatively correlated with phenotypic age (all P < 0.0001), while their low-moderate-high groupings and phenotypic age showed a dose-decreasing relationship (all P for trend < 0.0001). However, the relationship between health behavior scores and its three subgroups and biological age was not statistically significant (P and P for trend > 0.05). Health factor scores were negatively associated with biological age and high health factor scores had greater β estimates than low health factor scores. Furthermore, no interactions were found between health behavior score and health factor score with biological ageing (P for interaction > 0.05). (Additional file 1: Table S3) explored the relationships between health behavior scores and health factor scores with PhenoAgeAccel and BioAgeAccel and generally agreed with these results. Interestingly, although health behaviors and health factors were negatively related to phenotypic age and phenotypic age acceleration, health factors had larger β estimates. Additionally, the association of each of the LE8 scores with biological ageing was exploited (Additional file 1:Fig. S2), with blood glucose scores having larger β-estimates with phenotypic age and blood pressure scores with biological age, respectively. In conclusion, both health behaviors and health factors were negatively linked to biological ageing, while the negative association was stronger for health factors and greater for blood glucose and blood pressure among health factors.

**Table 3:** Weighted linear regression displaying the relationship between health behaviors score/health factors score and biological ageing

	Crude model		Model 1		Crude model		Model 1	
	β (95%CI)	P	β (95%CI)	P	β (95%CI)	P	β (95%CI)	P
	Phenotypic age				Biological age			
Health behaviors score	− 0.015 (− 0.040,0.011)	0.258	− 0.054 (− 0.061, − 0.047)	< 0.0001	0.039 (0.018,0.060)	< 0.001	0.007 (− 0.001, 0.014)	0.079
Classification								
Low (0–49)	ref		ref		ref		ref	
Moderate (50–79)	− 1.026 (− 2.233,0.181)	0.094	− 1.378 (− 1.766, − 0.991)	< 0.0001	1.131 (0.011,2.250)	0.048	0.711 ( 0.311, 1.110)	0.001
High (80–100)	− 0.942 (− 2.335,0.450)	0.18	− 2.624 (− 3.002, − 2.245)	< 0.0001	1.841 (0.699,2.984)	0.002	0.366 (− 0.081, 0.813)	0.104
p for trend		0.2		< 0.0001		0.002		0.178
Health factors score	− 0.496 (− 0.516, − 0.475)	< 0.0001	− 0.088 (− 0.099, − 0.076)	< 0.0001	− 0.538 (− 0.554, − 0.522)	< 0.0001	− 0.166 (− 0.173, − 0.159)	< 0.0001
Classification								
Low (0–49)	ref		ref		ref		ref	
Moderate (50–79)	− 10.791 (− 11.809, − 9.772)	< 0.0001	− 2.294 (− 2.748, − 1.841)	< 0.0001	− 12.225 (− 13.075, − 11.375)	< 0.0001	− 4.572 (− 4.967, − 4.177)	< 0.0001
High (80–100)	− 24.087 (− 25.286, − 22.889)	< 0.0001	− 3.894 (− 4.502, − 3.286)	< 0.0001	− 26.501 (− 27.455, − 25.547)	< 0.0001	− 7.762 (− 8.182, − 7.342)	< 0.0001
p for trend		< 0.0001		< 0.0001		< 0.0001		< 0.0001
Health behaviors score*Health factors score p for interaction				0.33				0.10

Crudel model: unadjusted model;

Model 1: Adjusted for age, sex, race, marital status, education, poverty-income ratio, alcohol using, hypertension, CVD, diabetes, and cancer

### Stratified analyses of LE8 with phenotypic age and biological age

**Fig. 1:**
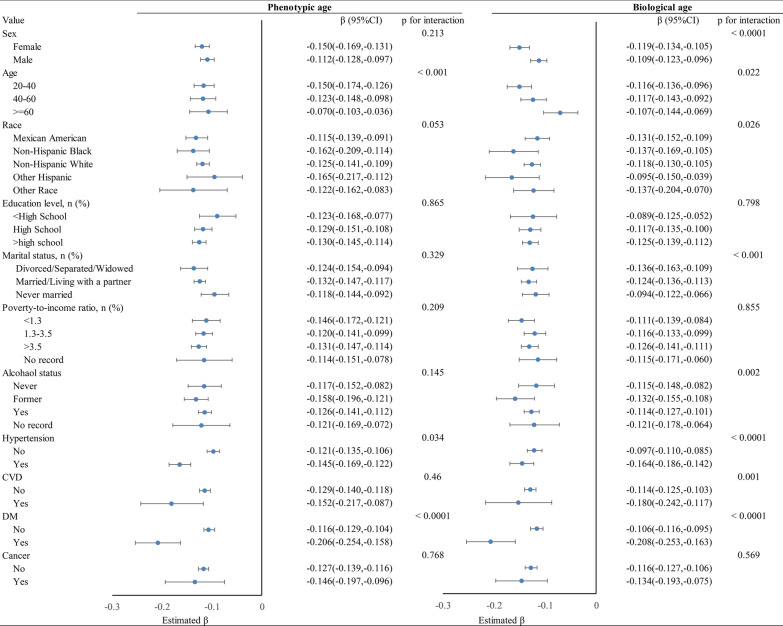
Stratified analyses on phenotypic age and biological age and LE8. *The analysis adjusted for age, sex, race, marital status, education, poverty-income ratio, alcohol using, hypertension, CVD, diabetes, and cancer. LE8, Life’s Essential 8

The stratified analyses, as illustrated in Fig. 1, indicated that LE8 had a strong negative correlation with phenotypic age and biological age. The negative association remained stable after stratification by sex, age, race, education, marital status, poverty, alcohol consumption status, hypertension, CVD, diabetes, and cancer.

### Restricted cubic spline analysis

**Fig. 2:**
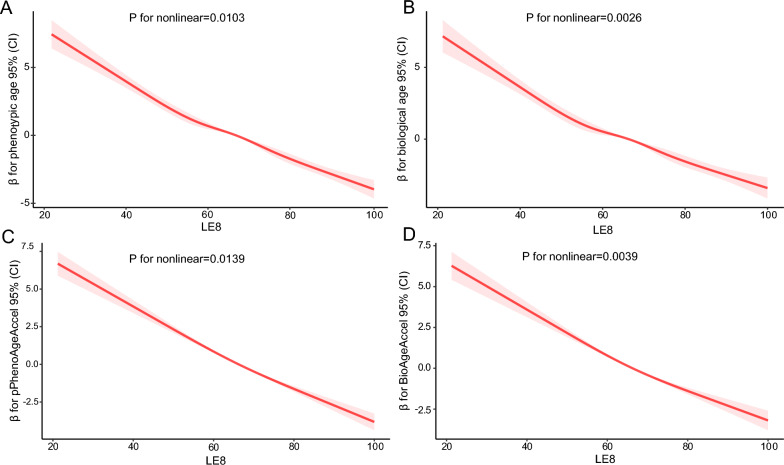
Analysis of Restricted Cubic Spline Regression. *Model adjusted for age, sex, race, marital status, education, poverty-income ratio, alcohol using, hypertension, CVD, diabetes, and cancer. LE8, Life’s Essential 8

In restricted cubic spline regressions adjusted for the covariates of interest, a significant nonlinear relationship between LE8 and biological ageing was uncovered (P for non-linear < 0.05, Fig. 2). Figures 2A–D demonstrated that phenotypic age, biological age, PhenoAgeAccel, and BioAgeAccel all decreased with increasing LE8, respectively.

## Discussion

To elucidate the relationship between LE8 scores and biological ageing, we carried out a cross-sectional analysis of 11,729 participants from the NHANES cohort. Negative associations were revealed between the LE8 score and its health behavior and health factor subscales and biological ageing. Stratified analyses illustrated that the inverse dose–response relationship between LE8 score and biological ageing remained stable across stratification factors. The association remained significant after excluding subjects with comorbidities. Intriguingly, health factors were more strongly negatively correlated with biological ageing, and blood glucose and blood pressure were more remarkable among the health factors.

Biological ageing is known to be assessed in a variety of ways and therefore several studies have evaluated the relationship between LS7 and biological ageing assessed using other modalities. From a study using 1999–2002 NHANES data, ideal cardiovascular health as calculated by LS7 was linked to longer leukocyte telomere length [18]. A cohort of postmenopausal women found that LS7 was negatively related to epigenetic age acceleration as measured by DNA methylation [19]. Hu et al. discovered that among participants with metabolic cardiovascular disease, the higher the LS7 score, the less phenotypic age acceleration and biological age acceleration [17]. Our findings are in general agreement with the above studies. However, since sleep is closely related to the health of the organism and LS7 lacks assessment of it, the definition of LS7 may not fully reflect the health behaviors and characteristics of the body than LS8.

In our study, the inverse relationship of LE8 and CVH with biological ageing was unchanged across populations. Ageing is affected by both genetic and epigenetic factors, with oxidative stress (OS) being the main epigenetic element based on Harman's free radical theory of ageing [20, 21]. In addition, the diverse mechanisms that drive the ageing process include macromolecular damage, disruption of proteostasis, inflammation and stem cell exhaustion, epigenetic drift, metabolic dysregulation, and dysregulation of the stress response. These processes are known as the "seven pillars of ageing" and are important manifestations of the biological ageing process [22, 23]. The progression of CVD is closely linked to ageing-related cells and molecules, particularly low-grade inflammation and oxidative stress that may damage the vascular endothelium triggering a range of pathological responses [24–26]. Therefore, LE8 and CVH may slow down ageing by improving many of these pathways.

Healthy behaviors including proper diet, physical activity, not smoking, and sleep, can prevent ageing. Dietary diversity is an important indicator of nutritional adequacy. Increasing dietary diversity improves dietary quality, ensures adequate nutrient intake, and promotes healthy ageing [27, 28]. Higher total dietary antioxidant capacity or dietary diversity in midlife and increased adherence to healthy eating patterns in mid- and late-life are related to healthy ageing in later life [29]. Numerous studies have shown that sticking to present physical activity guideline suggestions can decrease the risk of chronic diseases such as CVD and diabetes, and benefit overall health [30]. Physical activity, no matter the type of exercise, is useful in lowering inflammatory markers in older adults [31]. Nicotine, as an exogenous agonist of nicotinic acetylcholine receptors (nAChR) to modulate nAChR expression, leads to senescent changes by intervening in multiple pathological molecular pathways [32]. Unlike LS7, LE8 additionally included sleep indicators. Interestingly, good sleep was negatively associated with ageing, which is consistent with previous findings [33, 34]. Ageing is associated with longer sleep latency, more fragile sleep (increased arousals), shorter sleep duration, and reduced time spent in the slow-wave phase of non-rapid eye movement sleep [35]. Sleep contributes to the sleep-related activity of the lymphatic system—the removal of metabolic wastes—whereas sleep deprivation impairs waste removal and accelerates ageing [36]. In addition, sleep deprivation affects neurogenesis, long-term inhibition and long-term potentiation, dendritic spine structure and function, and synaptic plasticity, further causing ageing and cognitive decline [37].

Health factors comprising favorable BMI, blood lipids, blood sugar, and blood pressure help to slow down the ageing process. Several studies have confirmed that maintaining a normal BMI can prolong survival, increase healthy life expectancy, improve physical functioning in older adults, and slow ageing [38–40]. Plasma lipid metabolism is connected to indicators of ageing and healthy lifespan in healthy adults by affecting multiple molecular pathways and cell types [41–43]. Restoring aberrant lipid metabolism is a novel and promising strategy to combat ageing [44]. Blood pressure is often considered one of the most important modifiable vascular risk factors for preventing or delaying ageing and dementia [45]. Ageing is characterized by alterations in neuro-cardiovascular regulatory mechanisms leading to impaired patterns of physiological variability [46]. Alterations in ageing and blood pressure share many of the same molecular mechanisms, including subclinical inflammation, increased ROS production, altered endothelial function, arterial stiffness, autonomic dysfunction, genomic instability, mitochondrial oxidative damage, and epigenetic modifications, among others [46, 47]. In addition, hypertension is associated with cortical atrophy, particularly in the hippocampus and frontal cortical regions, thereby accelerating brain ageing [48]. Blood glucose is strongly correlated with ageing, and the majority of previous studies have focused on brain ageing. In healthy individuals, high blood glucose promotes brain atrophy, and the effects of blood glucose on the brain are not unique to type 2 diabetes, as blood glucose levels can have a significant impact on whole-brain and grey matter atrophy, even within the normal range [49–51]. Animal models of both type 1 and type 2 diabetes have demonstrated that chronic high blood glucose levels lead to impaired synaptic plasticity and accelerated cognitive deficits caused by brain ageing [52]. The specific mechanisms of blood glucose and systemic biological ageing will require further study in the future. In our study, health factors were found to be more associated with ageing than health behaviors, probably because health factors are directly associated with ageing, whereas health behaviors slow down ageing by improving health factors.

The present study has several strengths. First, our study used the more updated LE8 to reflect cardiovascular health, as well as analyzed the components of the LE8 with biological ageing, making the findings more comprehensive and targeted. Second, NHANES used a complex multistage probability sampling design to draw a representative civilian non-institutionalized resident population to ensure higher data quality. As a result, extrapolation of the results to the entire U.S. civilian non-institutionalized population has a high degree of reliability. Third, the present study conducted several stratified analyses and uncovered a solid association between LE8 and biological ageing in different populations, thus making the findings more generalizable. Thus, the findings have broader public health implications for the prevention of ageing.

The current study also has some limitations. Firstly, it is difficult to establish a causal relationship between LE8 and biological ageing as this study was cross-sectional. Therefore, more prospectively designed studies are needed to demonstrate the validity of the LE8. Second, the assessment of health behavior indicators was based on self-report questionnaires, which are susceptible to recall bias. Third, this study did not investigate biological ageing at the molecular or cellular level, but only used clinical markers such as phenotypic age and biological age. However, the use of two different methods of calculating biological ageing allowed the results of both to be corroborated with each other, which greatly increased the robustness of our findings.

## Conclusions

In this nationally representative sample of U.S. adults, LE8 scores, health behavior scores, and health factor scores were strongly negatively associated with biological ageing. Moreover, health factors were more significantly negatively correlated with biological ageing than health behaviors and blood glucose and blood pressure were more prominent among the health factors. The outcomes emphasize that LE8 may be an effective way to slow down ageing and that maintaining cardiovascular health may prevent biological ageing. And besides good lifestyle behaviors, reasonable blood pressure and blood glucose may be more crucial in preventing ageing. In the future, the causal relationship and exact mechanism between LE8 and biological ageing should be further explored.

### Supplementary Information

**Additional file 1:** Supplementary methods and results. **Figure S1.** Flowchart of the sample selection from NHANES 2005–2010. **Table S1**. Definition and scoring approach for the American Heart Association’s Life’s Essential 8 score. **Table S2.** Sensitivity analyses on LE8/CVH and biological ageing. **Table S3.**Weighted linear regression displaying the relationship between health behaviors score/health factors score and biological ageing. **Figure S2.** Weighted linear regressions on each of the LE8 items with biological ageing.

## Data Availability

Data from the National Health and Nutrition Examination Survey (NHANES) 1999–2018 are publicly available online (https://www.cdc.gov/nchs/nhanes/index.htm).

## Abbreviations

BMI: Body mass index
BioAgeAccel: Biological age acceleration
CI: Confidence interval
CVD: Cardiovascular disease
CVH: Cardiovascular health
DM: Diabetes mellitus
HEI: Healthy eating index
LE8: Life’s Essential 8
NHANES: National Health and Nutrition Examination Survey
PhenoAgeAccel: Phenotypic age acceleration

## References

[1] Zhang W, Peng S-F, Chen L, Chen H-M, Cheng X-E, Tang Y-H (2022). Association between the oxidative balance score and telomere length from the National Health and Nutrition Examination Survey 1999–2002. Oxid Med Cell Longev.

[2] Chakravarti D, LaBella KA, DePinho RA (2021). Telomeres: history, health, and hallmarks of aging. Cell.

[3] Kennedy BK, Berger SL, Brunet A, Campisi J, Cuervo AM, Epel ES (2014). Geroscience: linking aging to chronic disease. Cell.

[4] Hahad O, Frenis K, Kuntic M, Daiber A, Münzel T (2021). Accelerated aging and age-related diseases (CVD and Neurological) due to air pollution and traffic noise exposure. Int J Mol Sci.

[5] Roetker NS, Pankow JS, Bressler J, Morrison AC, Boerwinkle E (2018). Prospective study of epigenetic age acceleration and incidence of cardiovascular disease outcomes in the ARIC Study (Atherosclerosis Risk in Communities). Circ Genom Precis Med.

[6] Mwasongwe S, Gao Y, Griswold M, Wilson JG, Aviv A, Reiner AP (2017). Leukocyte telomere length and cardiovascular disease in African Americans: The Jackson Heart Study. Atherosclerosis.

[7] Huang R-C, Lillycrop KA, Beilin LJ, Godfrey KM, Anderson D, Mori TA (2019). Epigenetic Age Acceleration in Adolescence Associates With BMI, Inflammation, and Risk Score for Middle Age Cardiovascular Disease. J Clin Endocrinol Metab.

[8] Ammous F, Zhao W, Ratliff SM, Mosley TH, Bielak LF, Zhou X (2021). Epigenetic age acceleration is associated with cardiometabolic risk factors and clinical cardiovascular disease risk scores in African Americans. Clin Epigenetics.

[9] Lloyd-Jones DM, Hong Y, Labarthe D, Mozaffarian D, Appel LJ, Van Horn L (2010). Defining and setting national goals for cardiovascular health promotion and disease reduction: the American Heart Association’s strategic Impact Goal through 2020 and beyond. Circulation.

[10] Lloyd-Jones DM, Allen NB, Anderson CAM, Black T, Brewer LC, Foraker RE (2022). Life’s Essential 8: updating and enhancing the American Heart Association’s construct of cardiovascular health: a presidential advisory from the American Heart Association. Circulation.

[11] Lloyd-Jones DM, Ning H, Labarthe D, Brewer L, Sharma G, Rosamond W (2022). Status of cardiovascular health in US Adults and children using the american heart association’s new “Life’s Essential 8” metrics: prevalence estimates from the national health and nutrition examination survey (NHANES), 2013 through 2018. Circulation.

[12] Klemera P, Doubal S (2006). A new approach to the concept and computation of biological age. Mech Ageing Dev.

[13] Levine ME, Lu AT, Quach A, Chen BH, Assimes TL, Bandinelli S (2018). An epigenetic biomarker of aging for lifespan and healthspan. Aging (Albany NY).

[14] Hertel J, Friedrich N, Wittfeld K, Pietzner M, Budde K, Van der Auwera S (2016). Measuring biological age via metabonomics: the metabolic age score. J Proteome Res.

[15] Krebs-Smith SM, Pannucci TE, Subar AF, Kirkpatrick SI, Lerman JL, Tooze JA (2018). Update of the healthy eating index: HEI-2015. J Acad Nutr Diet.

[16] Levine ME (2013). Modeling the rate of senescence: can estimated biological age predict mortality more accurately than chronological age?. J Gerontol A Biol Sci Med Sci.

[17] Hu Y, Wang X, Huan J, Zhang L, Lin L, Li Y (2022). Effect of dietary inflammatory potential on the aging acceleration for cardiometabolic disease: a population-based study. Front Nutr.

[18] Gebreab SY, Manna ZG, Khan RJ, Riestra P, Xu R, Davis SK (2017). Less than ideal cardiovascular health is associated with shorter leukocyte telomere length: The National Health and Nutrition Examination Surveys, 1999–2002. J Am Heart Assoc.

[19] Pottinger TD, Khan SS, Zheng Y, Zhang W, Tindle HA, Allison M (2021). Association of cardiovascular health and epigenetic age acceleration. Clin Epigenetics.

[20] Chaudhary MR, Chaudhary S, Sharma Y, Singh TA, Mishra AK, Sharma S (2023). Aging, oxidative stress and degenerative diseases: mechanisms, complications and emerging therapeutic strategies. Biogerontology..

[21] Aunan JR, Watson MM, Hagland HR, Søreide K (2016). Molecular and biological hallmarks of ageing. Br J Surg.

[22] J G, E W, J S, Ab M, Bk K. Targeting the molecular & cellular pillars of human aging with exercise. The FEBS journal. 2023;290 (Acesed1 Aug 2023). 10.1111/febs.16337 34968001

[23] Klaips CL, Jayaraj GG, Hartl FU (2018). Pathways of cellular proteostasis in aging and disease. J Cell Biol.

[24] Man AWC, Li H, Xia N (2020). Impact of lifestyles (diet and exercise) on vascular health: oxidative stress and endothelial function. Oxid Med Cell Longev.

[25] Shih C-C, Shih Y-L, Chen J-Y (2021). The association between homocysteine levels and cardiovascular disease risk among middle-aged and elderly adults in Taiwan. BMC Cardiovasc Disord.

[26] Stakos DA, Stamatelopoulos K, Bampatsias D, Sachse M, Zormpas E, Vlachogiannis NI (2020). The alzheimer’s disease amyloid-beta hypothesis in cardiovascular aging and disease: JACC focus seminar. J Am Coll Cardiol.

[27] Zheng J, Zhou R, Li F, Chen L, Wu K, Huang J (2021). Association between dietary diversity and cognitive impairment among the oldest-old: findings from a nationwide cohort study. Clin Nutr.

[28] Zhang J, Zhao A (2021). Dietary diversity and healthy aging: a prospective study. Nutrients.

[29] Zhou Y-F, Song X-Y, Pan A, Koh W-P (2023). Nutrition and healthy ageing in Asia: a systematic review. Nutrients.

[30] Ungvari Z, Fazekas-Pongor V, Csiszar A, Kunutsor SK (2023). The multifaceted benefits of walking for healthy aging: from Blue Zones to molecular mechanisms. Geroscience..

[31] Khalafi M, Akbari A, Symonds ME, Pourvaghar MJ, Rosenkranz SK, Tabari E (2023). Influence of different modes of exercise training on inflammatory markers in older adults with and without chronic diseases: a systematic review and meta-analysis. Cytokine.

[32] Majdi A, Kamari F, Vafaee MS, Sadigh-Eteghad S (2017). Revisiting nicotine’s role in the ageing brain and cognitive impairment. Rev Neurosci.

[33] Zhang Y, Xia X, Zhang T, Zhang C, Liu R, Yang Y (2023). Relation between sleep disorders and post-stroke cognitive impairment. Front Aging Neurosci.

[34] Kusters CDJ, Klopack ET, Crimmins EM, Seeman TE, Cole S, Carroll JE (2023). Short sleep and insomnia are associated with accelerated epigenetic age. Psychosom Med..

[35] Mander BA, Winer JR, Walker MP (2017). Sleep and human aging. Neuron.

[36] Hablitz LM, Nedergaard M (2021). The glymphatic system: a novel component of fundamental neurobiology. J Neurosci.

[37] Am M, B R, S T, A B, M B, Sr B, et al. Sleep, brain vascular health and ageing. GeroScience. 2020;42. (Accessed 1 Aug 2023) 10.1007/s11357-020-00235-8 PMC7525637 32748314

[38] Holme I, Tonstad S (2015). Survival in elderly men in relation to midlife and current BMI. Age Ageing.

[39] Leigh L, Byles JE, Jagger C (2016). BMI and healthy life expectancy in old and very old women. Br J Nutr.

[40] Hajek A, König H-H (2017). The curvilinear effect of BMI on functional health-evidence of the long-running german ageing survey. Obes Facts.

[41] Johnson LC, Martens CR, Santos-Parker JR, Bassett CJ, Strahler TR, Cruickshank-Quinn C (2018). Amino acid and lipid associated plasma metabolomic patterns are related to healthspan indicators with ageing. Clin Sci (Lond).

[42] Johnson LC, Parker K, Aguirre BF, Nemkov TG, D’Alessandro A, Johnson SA (2019). The plasma metabolome as a predictor of biological aging in humans. Geroscience.

[43] Rubio-Tomás T, Tavernarakis N (2022). Lipid metabolism and ageing in Caenorhabditis elegans: a complex interplay. Biogerontology.

[44] Liu H-J, Miao H, Yang J-Z, Liu F, Cao G, Zhao Y-Y (2023). Deciphering the role of lipoproteins and lipid metabolic alterations in ageing and ageing-associated renal fibrosis. Ageing Res Rev.

[45] Livingston G, Huntley J, Sommerlad A, Ames D, Ballard C, Banerjee S (2020). Dementia prevention, intervention, and care: 2020 report of the Lancet Commission. The Lancet.

[46] Bencivenga L, De Souto BP, Rolland Y, Hanon O, Vidal J-S, Cestac P (2022). Blood pressure variability: a potential marker of aging. Ageing Res Rev.

[47] Donato AJ, Machin DR, Lesniewski LA (2018). Mechanisms of dysfunction in the aging vasculature and role in age-related disease. Circ Res.

[48] Gutteridge DS, Segal A, McNeil JJ, Beilin L, Brodtmann A, Chowdhury EK (2023). The relationship between long-term blood pressure variability and cortical thickness in older adults. Neurobiol Aging.

[49] Walsh EI, Shaw M, Sachdev P, Anstey KJ, Cherbuin N (2018). Brain atrophy in ageing: ESTIMATING effects of blood glucose levels vs. other type 2 diabetes effects. Diabet Metabol..

[50] Brundel M, Kappelle LJ, Biessels GJ (2014). Brain imaging in type 2 diabetes. Eur Neuropsychopharmacol.

[51] Shaw ME, Nettersheim J, Sachdev PS, Anstey KJ, Cherbuin N (2017). Higher fasting plasma glucose is associated with increased cortical thinning over 12 years: the PATH through life study. Brain Topogr.

[52] Artola A (2013). Diabetes mellitus- and ageing-induced changes in the capacity for long-term depression and long-term potentiation inductions: toward a unified mechanism. Eur J Pharmacol.

