# Supplementary material for: Association between life’s essential 8 and biological ageing among US adults
Source: J Transl Med. 2023 Sep 14;21:622. doi: 10.1186/s12967-023-04495-8 (PMC10503107; doi:10.1186/s12967-023-04495-8)
Supplement: Supplementary file 1 — Additional file 1: Supplementary methods and results. Figure S1. Flowchart of the sample selection from NHANES 2005–2010. Table S1. Definition and scoring approach for the American Heart Association’s Life’s Essential 8 score. Table S2. Sensitivity analyses on LE8/CVH and biological ageing. Table S3.Weighted linear regression displaying the relationship between health behaviors score/health factors score and biological ageing. Figure S2. Weighted linear regressions on each of the LE8 items with biological ageing. [file 12967_2023_4495_MOESM1_ESM.docx]

**Additional MATERIALS**

**Association between Life’s Essential 8 and Biological Ageing among US Adults**

Ronghuai Zhang ^1^, Min Wu ^2^, Wei Zhang ^1^, Xuna Liu ^3^, Jie Pu ^1^, Tao Wei ^1,4^, Zhanfang Zhu ^5^, Zhiguo Tang ^1^, Na Wei ^1^ , Bo Liu ^1^, Qianwei Cui ^1^, Junkui Wang ^1^, Fuqiang Liu ^1^, and Ying Lv ^1^*

^1^ Department of Cardiology, Shaanxi Provincial People's Hospital, Xi’an, P. R. China.

^2^ Shaanxi Provincial Key Laboratory of Infection and Immune Diseases, Shaanxi Provincial People's Hospital, Xi’an, P. R. China.

^3^ Department of Gastroenterology, The Second Affiliated Hospital of Xi’an Jiaotong University, Xi’an 710004, China

^4^ Department of cardiovascular surgery, Shaanxi Provincial People's Hospital, Xi’an, P. R. China.

^5^ Xi'an Jiaotong University Hospital, Xi’an, P. R. China.

^*^ Correspondence: yinglv0802@163.com; Tel: +86-15596809585

**^*^Corresponding author:**

Ying Lv, MD.

**Figure S1. Flowchart of the sample selection from NHANES 2005-2010**

Participants extracted from NHANES

2005-2010 (n=31034)

Excluded:

Participants with age <20 years (n=13902).

Included participants (n=17132)

Excluded:

Participants with missing data on Life’s Essential 8 (n=4229);

Included participants (n=12903)

Excluded:

Participants with missing values regarding phenotypic age and biological age (n = 1156).

Included participants (n=11747)

Eligible participants for analysis (n=11729)

Excluded:

Participants with missing data on marriage and education (n = 18);

**Table S1. Definition and scoring approach for the American Heart Association’s Life’s Essential 8 score.**

| Domain | CVH Metric | Measurement | Quantification and Scoring of CVH Metric |
| --- | --- | --- | --- |
| Health Behaviors | Diet | Healthy Eating Index-2015 diet score percentile | Quantiles of DASH-style diet adherence  **Scoring (Population):**  Points Quantile  100 ≥95^th^ percentile (top/ideal diet)  80 75^th^ – 94^th^ percentile  50 50^th^ – 74^th^ percentile  25 25^th^ – 49^th^ percentile  0 1^st^ – 24^th^ percentile (bottom/least ideal quartile) |
|  | Physical activity | Self-reported minutes of moderate or vigorous physical activity per week | **Metric:** Minutes of moderate (or greater) intensity activity per week  **Scoring:**  Points Minutes  100 ≥150  90 120 – 149  80 90 – 119  60 60 – 89  40 30 – 59  20 1 – 29  0 0 |
|  | Nicotine exposure | Self-reported use of cigarettes or inhaled nicotine- delivery system | **Metric:** Combustible tobacco use and/or inhaled NDS use; or secondhand smoke exposure  **Scoring:**  Points Status  100 Never smoker  75 Former smoker, quit ≥5 yrs  50 Former smoker, quit 1 - <5 yrs  25 Former smoker, quit <1 year, or currently using inhaled NDS  0 Current smoker  Subtract 20 points (unless score is 0) for living with active indoor smoker in home |
|  | Sleep health | Self-reported average hours of sleep per night | **Metric:** Average hours of sleep per night  **Scoring:**  Points Level  100 7 – <9  90 9 – <10  70 6 – <7  40 5 – <6 or ≥10  20 4 – <5  0 <4 |
| Health Factors | Body mass index | Body weight (kg) divided by height squared (m^2^) | **Metric:** Body mass index (kg/m^2^)  **Scoring:** Points Level 100 <25  70 25.0 – 29.9  30 30.0 – 34.9  15 35.0 – 39.9  0 ≥40.0 |
|  | Blood lipids | Plasma total and HDL-cholesterol with calculation of non-HDL-cholesterol | **Metric:** Non-HDL-cholesterol (mg/dL)  **Scoring:**  Points Level  100 <130  60 130 – 159  40 160 – 189  20 190 – 219  0 ≥220  If drug-treated level, subtract 20 points |
|  | Blood glucose | Fasting blood glucose or casual hemoglobin A1c | **Metric:** Fasting blood glucose (mg/dL) or Hemoglobin A1c (%)  **Scoring:**  Points Level  100 No history of diabetes and FBG <100 (or HbA1c < 5.7)  60 No diabetes and FBG 100 – 125 (or HbA1c 5.7-6.4) (Pre-diabetes)  40 Diabetes with HbA1c <7.0  30 Diabetes with HbA1c 7.0 – 7.9  20 Diabetes with HbA1c 8.0 – 8.9  10 Diabetes with Hb A1c 9.0 – 9.9  0 Diabetes with HbA1c ≥10.0 |
|  | Blood pressure | Appropriately measured systolic and diastolic blood pressure | **Metric:** Systolic and diastolic blood pressure (mm Hg)  **Scoring:**  Points Level  100 <120/<80 (Optimal)  75 120-129/<80 (Elevated)  50 130-139 or 80-89 (Stage I HTN)  25 140-159 or 90-99  0 ≥160 or ≥100  Subtract 20 points if treated level |

**Reference**

1. Lloyd-Jones DM, Allen NB, Anderson CAM, et al. Life's Essential 8: Updating and Enhancing the American Heart Association's Construct of Cardiovascular Health: A Presidential Advisory From the American Heart Association. *Circulation*. Aug 2 2022;146(5):e18-e43.
2. Lloyd-Jones DM, Ning H, Labarthe D, et al. Status of Cardiovascular Health in US Adults and Children Using the American Heart Association's New "Life's Essential 8" Metrics: Prevalence Estimates From the National Health and Nutrition Examination Survey (NHANES), 2013 Through 2018. *Circulation*. Sep 13 2022;146(11):822-835.

**Table S2.** **Sensitivity analyses on LE8/CVH and biological ageing.**

|  | Crude model | | Model 1 | |
| --- | --- | --- | --- | --- |
|  | β (95%CI) | P | β (95%CI) | P |
|  | **Phenotypic age** | | | |
| LE8 | -0.231(-0.272,-0.189) | <0.0001 | -0.118(-0.131,-0.105) | <0.0001 |
| CVH |  |  |  |  |
| Low | ref |  | ref |  |
| Moderate | -5.778( -8.071,-3.484) | <0.0001 | -2.344(-3.309,-1.379) | <0.0001 |
| High | -10.495(-13.103,-7.887) | <0.0001 | -4.534(-5.467,-3.602) | <0.0001 |
| p for trend |  | <0.0001 |  | <0.0001 |
|  |  |  |  |  |
|  | **Biological age** | | | |
| LE8 | -0.195(-0.225,-0.164) | <0.0001 | -0.092(-0.106,-0.078) | <0.0001 |
| CVH |  |  |  |  |
| Low | ref |  | ref |  |
| Moderate | -4.707( -6.635,-2.780) | <0.0001 | -1.996(-2.899,-1.094) | <0.0001 |
| High | -8.729(-10.874,-6.583) | <0.0001 | -3.618(-4.607,-2.628) | <0.0001 |
| p for trend |  | <0.0001 |  | <0.0001 |

Crudel model: unadjusted model;

Model 1: Adjusted for age, sex, race, marital status, education, poverty-income ratio, and alcohol using.

LE8, life’s essential 8; CVH, cardiovascular health;

Low CVH was defined as a LE8 score of 0 to 49, moderate CVH of 50–79, and high CVH of 80–100.

**Table S3.Weighted linear regression displaying the relationship between health behaviors score/health factors score and biological ageing.**

|  | Crude model | | | | Model 1 | | |  | Crude model | | | Model 1 | | |
| --- | --- | --- | --- | --- | --- | --- | --- | --- | --- | --- | --- | --- | --- | --- |
|  | β (95%CI) | | P | | β (95%CI) | P | |  | β (95%CI) | P | | β (95%CI) | P | |
|  | | **PhenoAgeAccel** | | | | | |  | **BioAgeAccel** | | | | | |
| Health behaviors score | | -0.078(-0.085,-0.070) | | <0.0001 | -0.054(-0.061,-0.047) | | <0.0001 |  | -0.024(-0.032,-0.016) | | <0.0001 | 0.007(-0.001, 0.014) | | 0.079 |
| Classification | |  | |  |  | |  |  |  | |  |  | |  |
| Low (0–49) | | ref | |  | ref | |  |  | ref | |  | ref | |  |
| Moderate (50–79) | | -2.119(-2.494,-1.744) | | <0.0001 | -1.378(-1.766,-0.991) | | <0.0001 |  | 0.038(-0.365, 0.440) | | 0.852 | 0.711( 0.311, 1.110) | | 0.001 |
| High (80–100) | | -3.955(-4.359,-3.550) | | <0.0001 | -2.624(-3.002,-2.245) | | <0.0001 |  | -1.171(-1.580,-0.762) | | <0.0001 | 0.366(-0.081, 0.813) | | 0.104 |
| p for trend | |  | | <0.0001 |  | | <0.0001 |  |  | | <0.0001 |  | | 0.178 |
|  | |  | |  |  | |  |  |  | |  |  | |  |
| Health factors score | | -0.133(-0.141,-0.124) | | <0.0001 | -0.088(-0.099,-0.076) | | <0.0001 |  | -0.175(-0.184,-0.166) | | <0.0001 | -0.166(-0.173,-0.159) | | <0.0001 |
| Classification | |  | |  |  | |  |  |  | |  |  | |  |
| Low (0–49) | | ref | |  | ref | |  |  | ref | |  | ref | |  |
| Moderate (50–79) | | -4.659(-5.086,-4.233) | | <0.0001 | -2.294(-2.748,-1.841) | | <0.0001 |  | -6.094(-6.526,-5.661) | | <0.0001 | -4.572(-4.967,-4.177) | | <0.0001 |
| High (80–100) | | -7.024(-7.531,-6.517) | | <0.0001 | -3.894(-4.502,-3.286) | | <0.0001 |  | -9.437(-9.922,-8.953) | | <0.0001 | -7.762(-8.182,-7.342) | | <0.0001 |
| p for trend | |  | | <0.0001 |  | | <0.0001 |  |  | | <0.0001 |  | | <0.0001 |

Crudel model: unadjusted model;

Model 1: Adjusted for age, sex, race, marital status, education, poverty-income ratio, alcohol using, hypertension, CVD, diabetes, and cancer.

PhenoAgeAccel, phenotypic age acceleration; BioAgeAccel, biological age acceleration.

**Figure S2:** **Weighted linear regressions on each of the LE8 items with biological ageing.**


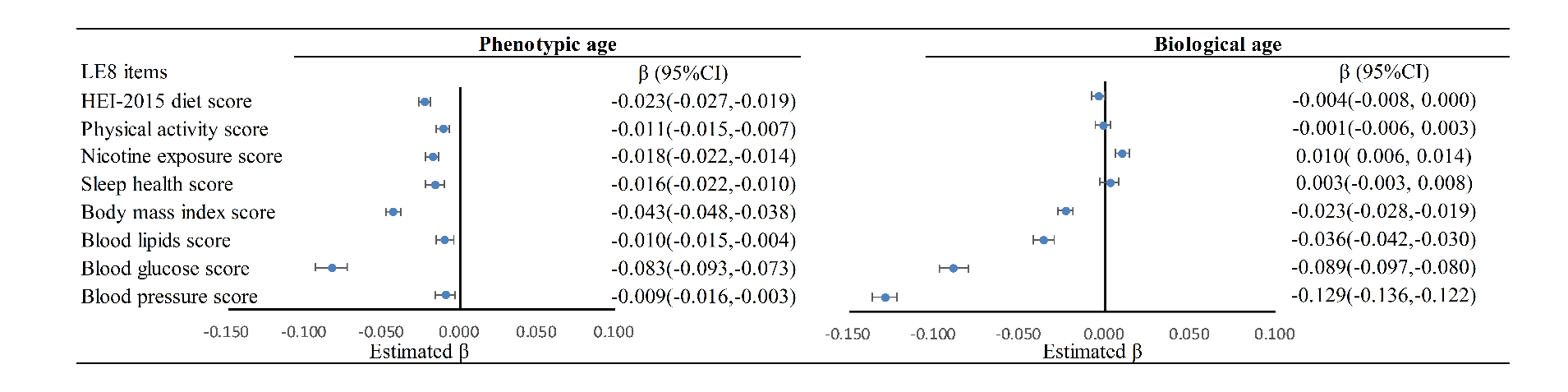


Model adjusted for age, sex, race, marital status, education, poverty-income ratio, alcohol using, hypertension, CVD, diabetes, and cancer.

LE8, Life’s Essential 8
